# Prevalent *de novo *somatic mutations in superantigen genes of mouse mammary tumor viruses in the genome of C57BL/6J mice and its potential implication in the immune system

**DOI:** 10.1186/1471-2172-12-5

**Published:** 2011-01-18

**Authors:** Young-Kwan Lee, Sophia Chiu, Alex Chew, David G Greenhalgh, Kiho Cho

**Affiliations:** 1 Shriners Hospitals for Children Northern California and Department of Surgery, University of California, Davis, Sacramento, CA 95817, USA

## Abstract

**Background:**

Superantigens (SAgs) of mouse mammary tumor viruses (MMTVs) play a crucial role in T cell selection in the thymus in a T cell receptor (TCR) Vβ-specific manner and SAgs presented by B cells activate T cells in the periphery. The peripheral T cell repertoire is dynamically shaped by the steady induction of T cell tolerance against self antigens throughout the lifespan. We hypothesize that *de novo *somatic mutation of endogenous MMTV SAgs contributes to the modulation of the peripheral T cell repertoire.

**Results:**

SAg coding sequences were cloned from the genomic DNAs and/or cDNAs of various tissues of female C57BL/6J mice. A total of 68 unique SAg sequences (54 translated sequences) were identified from the genomic DNAs of liver, lungs, and bone marrow, which are presumed to harbor only three endogenous MMTV loci (*Mtv-8*, *Mtv-9*, and *Mtv-17*). Similarly, 69 unique SAg sequences (58 translated sequences) were cloned from the cDNAs of 18 different tissues. Examination of putative TCR Vβ specificity suggested that some of the SAg isoforms identified in this study have Vβ specificities different from the reference SAgs of *Mtv-8*, *Mtv-9*, or *Mtv-17*.

**Conclusion:**

The pool of diverse SAg isoforms, generated by *de novo *somatic mutation, may play a role in the shaping of the peripheral T cell repertoire including the autoimmune T cell population.

## Background

Endogenous retroviruses (ERVs) are known to make up approximately 10 % of the mouse genome [1]. The available data suggest that the majority of the ERV population in the genome of C57BL/6J harbor sequences similar to murine leukemia viruses (MLVs). Limited copies of endogenous mouse mammary tumor viruses (MMTVs) are identified in the genome of almost all laboratory mouse strains, including C57BL/6J with three genomic loci of *Mtv-8*, *Mtv-9*, and *Mtv-17 *[2-4]. Although only three loci of endogenous MMTVs (*Mtv-8*, *Mtv-9*, and *Mtv-17*) are confirmed in the National Center for Biotechnology Information (NCBI) database, identification of the *Mtv-30 *superantigen (SAg) sequence from C57BL/6J mice has been reported [5]. Certain endogenous MMTVs, such as *Mtv-2*, are known to be capable of producing infectious virus particles, predominantly in the mammary gland, which are transmitted to the pups through the milk [6-9].

Both endogenous and exogenous MMTVs encode SAgs from an open reading frame residing on the 3' long terminal repeat (LTR) [10,11]. MMTV SAgs, which are type II membrane proteins presented in a major histocompatibility complex class II restricted manner, are capable of activating a large fraction of T cells via interaction with specific Vβ region(s) of T cell receptors (TCRs) [11-13]. Individual MMTV SAg isoforms display differential TCR Vβ specificities. During thymic T cell development, endogenous MMTV SAgs are recognized as self-antigens resulting in the clonal deletion of specific TCR Vβ T cell subsets [10,14-19]. In addition, presentation of MMTV SAgs in the peripheral immune system leads to the activation of TCR Vβ-specific T cell subsets followed by anergy and cell death [20-22]. Presumably, SAgs from exogenous MMTVs participate in the peripheral modulation of the T cell repertoire in a TCR Vβ-specific manner. However, it may be reasonable to speculate that altered forms of SAgs originating from endogenous MMTVs will acquire different binding affinities for the same Vβ chain and/or new TCR Vβ specificity. As a result, they may contribute to the dynamic shaping of the peripheral T cell repertoire by tolerance induction throughout the lifespan of the animal. In addition, various types of stress signals (e.g., hormone) are likely to increase the rate of MMTV SAg somatic mutation in mice leading to an altered post-stress peripheral T cell profile, which may then contribute to phenotypic variations in inbred laboratory animals.

In this study, we tested the hypothesis that a set of *de novo *somatic mutations in the endogenous MMTV SAg genes contribute to the dynamic shaping of the peripheral T cell repertoire by examining the presence of divergent SAg isoform profiles at the genome and expression levels.

## Results and Discussion

### *de novo *somatic mutations in endogenous MMTV SAg coding sequences

To examine the spectrum of *de novo *somatic mutation events in the endogenous MMTV SAg coding sequences in C57BL/6J mice, SAg sequences were PCR amplified and cloned from the genomic DNAs isolated from the liver, lungs, and bone marrow of normal mice. Liver and lungs were selected to represent differentiated tissues while bone marrow consists of both immature and mature (e.g., antibody-producing plasma cells) immune cells [23,24].

**Figure 1:**
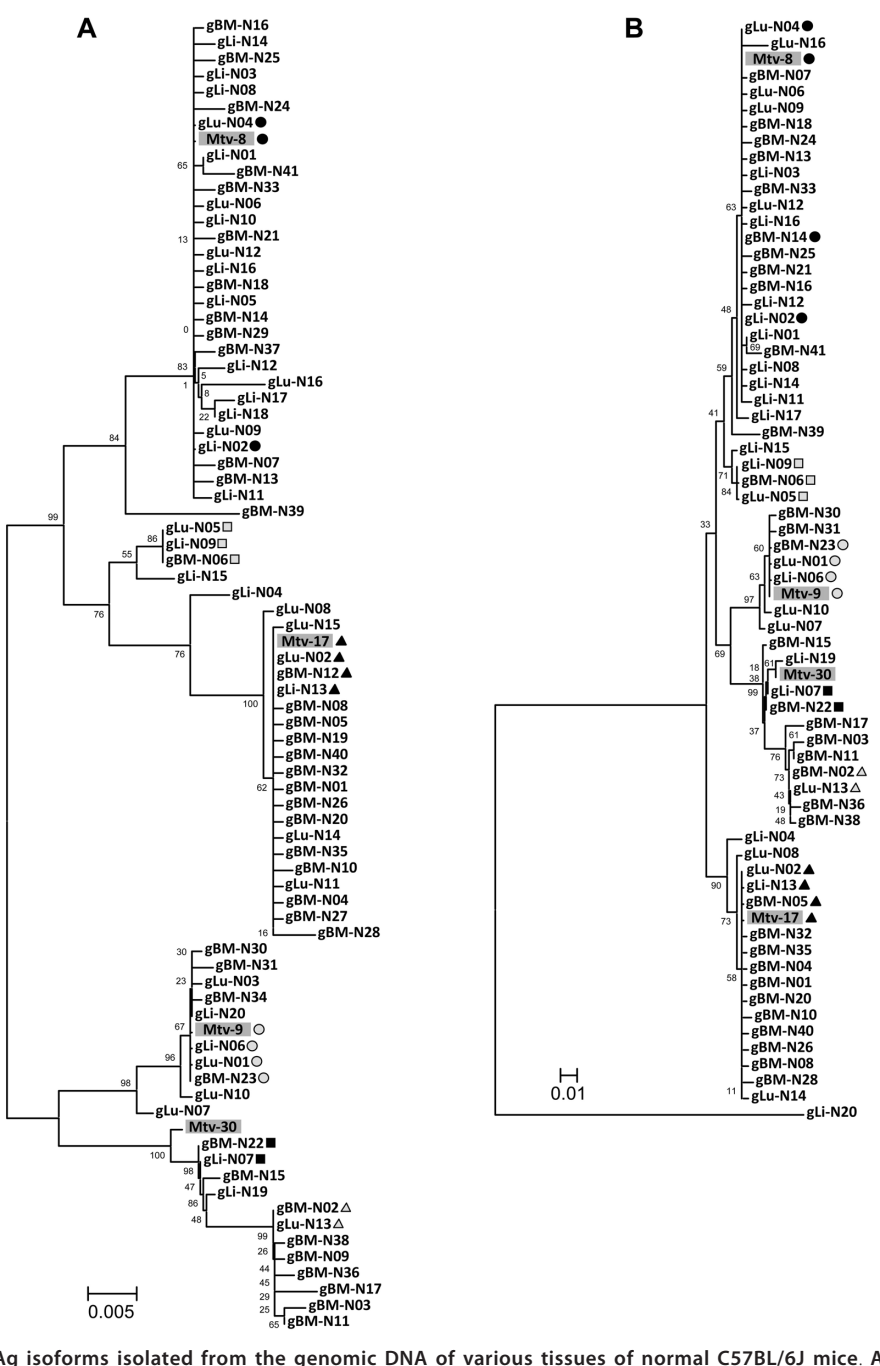
**MMTV SAg isoforms isolated from the genomic DNA of various tissues of normal C57BL/6J mice**. **A**. Phylogenetic tree (nucleotide sequence) of MMTV SAg isoforms. The three sets of unique MMTV SAg isoforms, which were isolated from the liver, lungs, and bone marrow, were phylogenetically analyzed. A number of unique branching units of SAg isoforms were formed in the phylogenetic tree. Unique SAg isoforms that were found in more than one tissue type are indicated using various shapes and shades. Four reference SAg sequences from *Mtv-8*, *Mtv-9*, *Mtv-17*, and *Mtv-30 *were included for this analysis. **B**. Phylogenetic tree (putative amino acid sequence) of SAg isoforms. The three sets (liver, lungs, and bone marrow) of unique SAg isoforms were analyzed and a phylogenetic tree with a number of unique branching units of SAg isoforms was formed. Unique SAg isoforms that were found in more than one tissue type are indicated using various shapes and shades. Four reference SAg sequences from *Mtv-8*, *Mtv-9*, *Mtv-17*, and *Mtv-30 *were included for this analysis. The translated sequence of gLi-N18 was not included in the analysis due to its short length resulting from a premature stop codon. black circle (identical to *Mtv-8*); black triangle (identical to *Mtv-17*); gray circle (identical to *Mtv-9*); gray triangle, gray box, and black box (identical sequences among different tissue types). gLi (liver genomic DNA), gLu (lung genomic DNA), gBM (bone marrow genomic DNA), N (normal tissue)

Alignment analyses of the SAg clones identified a number of unique SAg coding sequences within each tissue sample (nucleotide sequences/*in silico *translated amino acid sequences; 20/17 from liver, 16/13 from lungs, and 41/34 from bone marrow) (Figure 1). Some SAg coding sequences (nucleotide) were shared by more than one tissue and a total of 68 unique sequences were identified. A range of point mutations were observed in random loci throughout the SAg coding region. In some cases, mutations in SAg isoforms introduced a premature stop codon. Phylogenetic evaluation of the unique SAg coding sequences from all three tissues with the reference SAg sequences from *Mtv-8*, *Mtv-9*, *Mtv-17*, and *Mtv-30 *revealed a tree with a number of branching units. As expected, three branching units with the SAg reference sequences from *Mtv-8*, *Mtv-9*, and *Mtv-17*, which are the three endogenous MMTVs of C57BL/6J mice, had more SAg isoforms than the other branching units. Interestingly, the SAg isoforms in one branching unit were phylogenetically closer to the *Mtv-30 *SAg reference rather than to the SAgs from *Mtv-8*, *Mtv-9*, and *Mtv-17*. Furthermore, a substantial number of SAg clones formed unique branching units separate from any of the reference SAgs. A different, but somewhat similar branching pattern was observed within the phylogenetic tree of the *in silico *translated sequences (amino acid) of the SAg isoforms (Figure 1B).

The results presented in Figure 1 demonstrate that there are a high number of MMTV SAg isoforms in the genome of C57BL/6J mice, which had been previously reported to harbor only three endogenous MMTV loci (*Mtv-8*, *Mtv-9*, and *Mtv-17*) [2,4]. The presence of numerous MMTV SAg isoforms might represent a heterogeneous mixture (related but divergent) of mutated SAgs, derived from both endogenous and/or exogenous MMTV genomes in conjunction with a relatively high number of mutation events during the viral replication process [25]. Although *Mtv-8*, *Mtv-9*, and *Mtv-17 *are presumed to be inactive in replication, it is possible that biologically active viruses can be derived from recombination with replicating exogenous MMTVs in conjunction with the generation of numerous mutations in the SAg coding sequences [26]. For instance, a replication-competent recombinant MMTV provirus (5' LTR, *gag *and *pol *genes from a replicating *Mtv-2 *plus the *env *gene and 3' LTR from *Mtv-17*) has been reported in GR mice [27]. It has been described that exogenous MMTVs are able to infect and develop mammary tumors in C57BL/6J mice [28]. Mutations from the reverse transcription process of the retroviral RNA genome is reported to incur at an estimated rate of 0.05-1 mutation/genome/cycle. These *de novo *mutations from replicating MMTVs may occur constantly throughout the lifespan of the host. These findings provide some evidence suggesting that MMTVs replicate and mutate in C57BL/6J mice. Although the origins (endogenous and/or exogenous) of the proviral copies identified in this study are unknown, we noticed that three main branching units were formed along with the SAg references from *Mtv-8*, *Mtv-9*, and *Mtv-17*. Furthermore, the successful isolation and cloning of a number of SAg isoforms from genomic DNA suggests the integration of proviral copies of these MMTV isoforms into the genome of certain host cells.

### Expression of divergent MMTV SAg isoforms in various tissues

**Figure 2:**
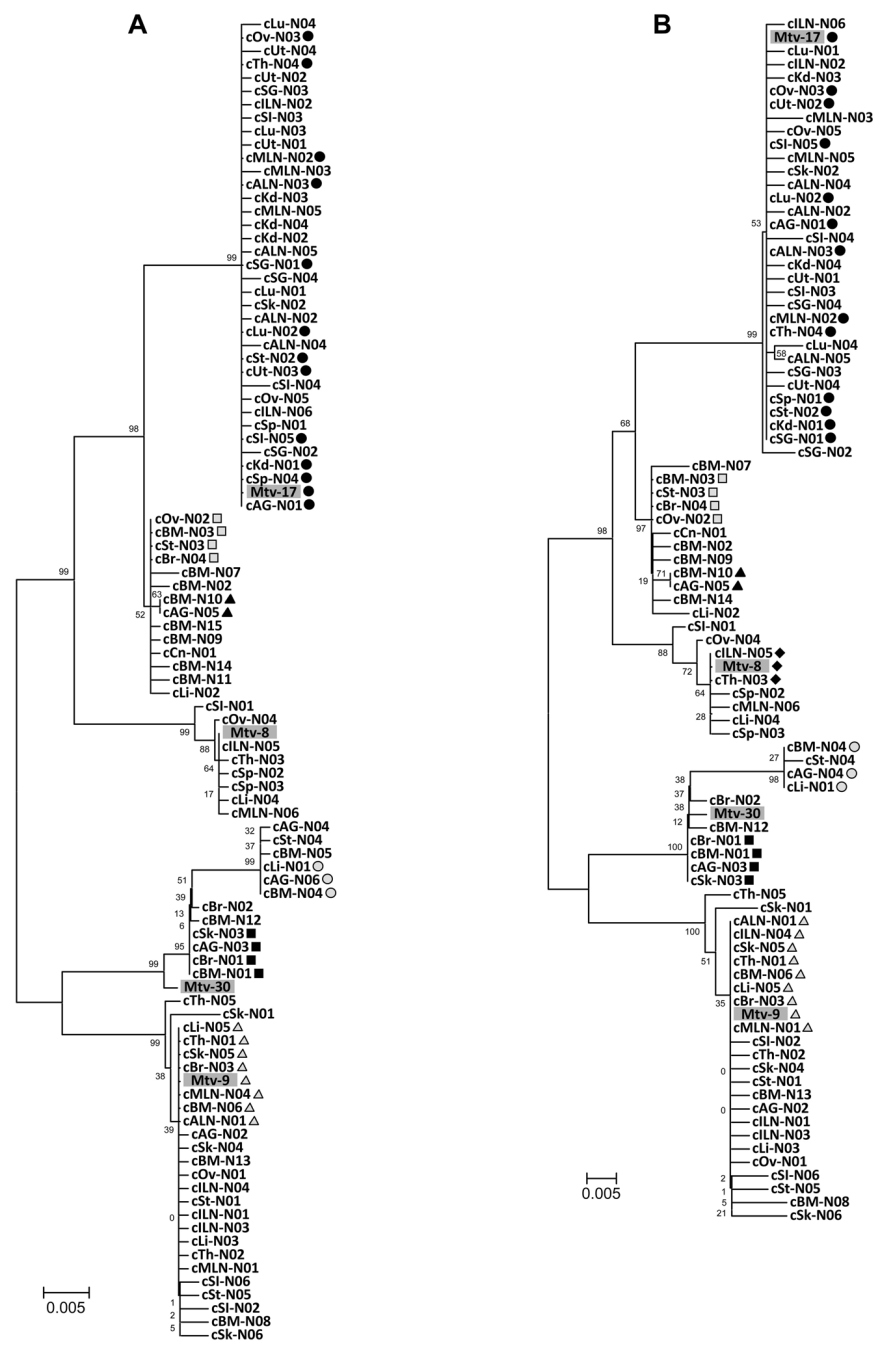
**MMTV SAg isoforms isolated from the cDNA from 18 different tissues of normal C57BL/6J mice**. **A**. Phylogenetic tree (nucleotide sequence) of MMTV SAg isoforms. Eighteen sets of unique MMTV SAg isoforms, which were isolated from 18 different tissues of normal C57BL/6J mice, were analyzed for their phylogenetic relatedness. A number of branching units of SAg isoforms were formed in the phylogenetic tree. Unique SAg isoforms that were found in more than one tissue type are indicated using various shapes and shades. Four reference SAg sequences from *Mtv-8*, *Mtv-9*, *Mtv-17*, and *Mtv-30 *were included for this analysis. **B**. Phylogenetic tree (putative amino acid sequence) of SAg isoforms. Eighteen sets of unique SAg isoforms were phylogenetically evaluated and a phylogenetic tree with a number of unique branching units was formed. Unique SAg isoforms that were found in more than one tissue type are indicated using various shapes and shades. Four reference SAg sequences from *Mtv-8*, *Mtv-9*, *Mtv-17*, and *Mtv-30 *were included for this analysis. black circle (identical to *Mtv-17*); gray triangle (identical to *Mtv-9*); black diamond (identical to *Mtv-8*); gray circle, black triangle, gray box, and black box (identical sequences among different tissue types). cLu (lung cDNA), cOv (ovary cDNA), cUt (uterus cDNA), cTh (thymus cDNA), cSG (salivary gland cDNA), cILN (inguinal lymph node cDNA), cSI (small intestine cDNA), cMLN (mesenteric lymph node cDNA), cALN (axillary lymph node cDNA), cKd (kidney cDNA), cSk (skin cDNA), cSt (stomach cDNA), cSp (spleen cDNA), cAG (adrenal gland cDNA), cBM (bone marrow cDNA), cBr (brain cDNA), cLi (liver cDNA), cCn (colon cDNA), N (normal tissue)

The identification of a number of MMTV SAg isoforms at the genomic level led us to investigate whether such a variability of SAg isoforms is present at the expression/transcription level and whether their mutation rates and profiles are associated with differences in tissue type. The SAg sequences were PCR cloned from cDNAs prepared from 18 different tissues from normal C57BL/6J mice (bone marrow, liver, lungs, kidney, salivary gland, adrenal gland, ovary, uterus, spleen, thymus, mesenteric lymph node, axillary lymph node, inguinal lymph node, small intestine, colon, brain, skin, and stomach) and subjected to alignment analyses to identify unique SAg coding sequences within each tissue. Subsequently, phylogenetic analyses of the SAg sequences from all 18 tissues (95 sequences in total) were performed to examine the distribution and similarities of the SAg cDNA isoforms (Figure 2A). Six SAg cDNA sequences were shared by more than one tissue type and a total of 69 unique SAg isoforms were identified from this study. The total number of unique SAg cDNA coding sequences (69) was similar to the number of unique genomic SAg coding sequences (68). However, a direct comparison of the number of unique SAg isoforms between these two groups (genomic vs. cDNA) may not be feasible since the cloning process was not normalized. Phylogenetic evaluation of the SAg cDNA isoforms with the same references used for the analysis of genomic SAg sequences revealed a unique tree pattern with a number of branching units that was substantially different from the genomic SAg tree (Figure 1A). A smaller number of SAg cDNA isoforms were present in the branching unit with the *Mtv-8 *SAg reference compared to the genomic SAg tree. In contrast, the branching unit with the *Mtv-9 *SAg reference had a larger number of SAg cDNA isoforms than the *Mtv-9 *branching unit in the genomic SAg tree. Similar to the genomic SAg tree, a few unique branching units, which are distant from the reference SAgs (*Mtv-8*, *Mtv-9*, *Mtv-17*, and *Mtv-30*), were formed in the SAg cDNA tree. One interesting finding is that none of the SAg cDNA isoforms isolated from the bone marrow were present in the branching units formed with the reference SAg of *Mtv-8 *or *Mtv-17*. The branching pattern of the SAg tree using *in silico *translated amino acid sequences resembled its nucleotide (cDNA) sequence tree (Figure 2B). Fifty eight unique SAg isoforms (translated amino acids) were identified in different tissues and a number of SAg cDNA isoforms share amino acid sequences that are identical to the reference *Mtv-8, -9 *and -*17 *SAgs. The unique branching pattern of the SAg cDNA isoforms (Figure 2) compared to the pattern from the genomic SAg isoforms (Figure 1) indicate that the expression of certain SAg isoforms is tissue type specific in conjunction with a range of internal as well as external stress signals. No significant differences in mutation rates were observed between the hypervariable and non-hypervariable regions of the SAgs at both the genomic DNA and cDNA levels.

### Examination of putative TCR Vβ specificity of SAg cDNA isoforms isolated from various tissues

**Figure 3:**
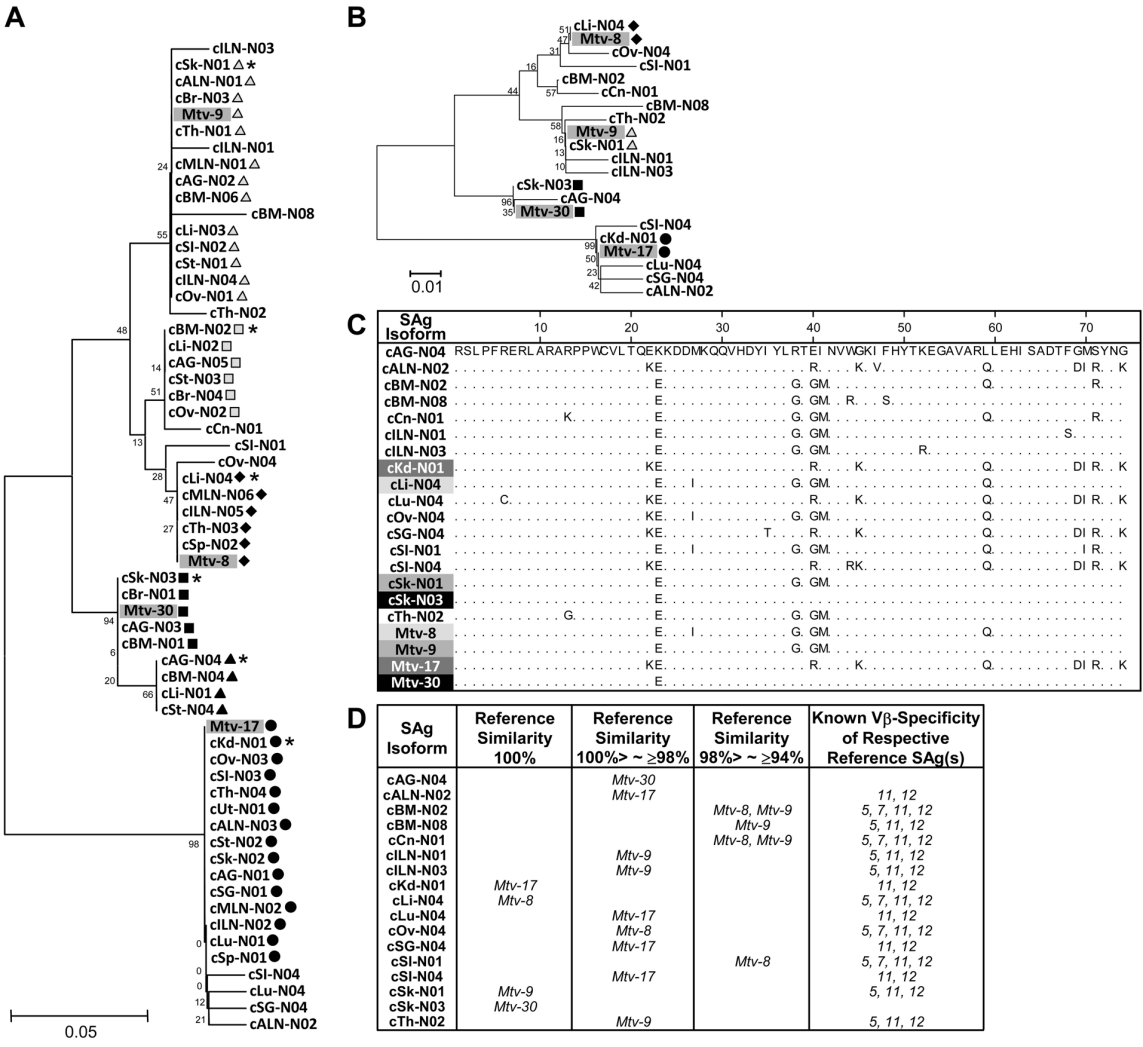
**Comparison of the hypervariable regions of MMTV SAg cDNA isoforms and their putative TCR Vβ specificity**. **A**. Phylogenetic tree of the C-terminus hypervariable regions of 56 SAg cDNA isoforms isolated from 18 different tissues. The C-terminus hypervariable regions (~74 amino acids) of the 56 SAg cDNA isoforms were phylogenetically analyzed. The C-terminus sequences that were found in more than one tissue type are indicated using various shapes and shades: black diamond (identical to *Mtv-8*); gray triangle (identical to *Mtv-9*); black circle (identical to *Mtv-17*); black box (identical to *Mtv*-30); gray box and black triangle (sequences [non-reference] shared among different tissues). * indicates a representative C-terminus sequence shared among different tissues. **B**. Phylogenic relatedness of 17 unique C-terminus sequences selected from the 56 SAg isoforms, which were isolated from 18 different tissues. Four reference C-terminus sequences from *Mtv-8*, *Mtv-9*, *Mtv-17*, and *Mtv-30 *are highlighted with gray. black diamond (identical to *Mtv-8*); gray triangle (identical to *Mtv-9*); black circle (identical to *Mtv-17*); black box (identical to *Mtv*-30). **C. **Comparison of the C-terminus hypervariable regions of 17 SAg isoforms. The unique C-terminus sequences of 17 SAg isoforms were compared with ones from four reference SAg sequences of *Mtv-8*, *Mtv-9*, *Mtv-17*, and *Mtv-30*. The SAg isoforms, identical to the individual reference SAgs, are indicated using various gray shades. **D**. Putative TCR Vβ specificity of SAg isoforms. Divergence of the SAg isoforms in regard to their putative TCR Vβ specificity was estimated by comparison with the TCR Vβ specificity of four reference SAg sequences from *Mtv-8*, *Mtv-9*, *Mtv-17*, and *Mtv-30*.

In this study, to determine whether changes in the hypervariable C-terminus regions of the SAg isoforms, which are known to determine the TCR Vβ specificity, affect their superantigenic function, we examined the putative TCR Vβ specificity of the SAg cDNA isoforms isolated from various tissues. Initially, unique C-terminus sequences (~74 amino acids) of individual SAg cDNA isoforms were selected within each tissue, and a total of 56 sequences were identified from all 18 tissues (Figure 3A). A phylogenetic analysis of the 56 C-terminus sequences identified 17 unique hypervariable region sequences (Figure 3B). Then, the 17 SAg C-terminus sequences were subjected to alignment analyses to identify the regions responsible for determining their putative TCR Vβ specificities (Figure 3C). The matching C-terminus sequences of the *Mtv-8*, *Mtv-9*, *Mtv-17*, and *Mtv-30 *SAgs and their reported TCR Vβ specificities were used as references [5,20,29,30]. Among the 17 unique C-terminus sequences, only four of them were 100 % homologous to the *Mtv-8*, *Mtv-9*, *Mtv-17*, or *Mtv-30 *SAg (Figure 3C/D).

The C-terminal ~74 amino acid region has been used as a reference for the classification of MMTV SAgs into seven families in regard to their TCR Vβ specificities [20,31]. These C-terminus regions from different MMTV SAgs are highly polymorphic. A region within the C-terminus (amino acid positions 42-74; Figure 3C) was determined to be important for TCR Vβ specificity, including binding affinity [32,33]. Among the 17 unique SAg isoforms, 13 SAgs had non-synonymous point mutations in this region in comparison to the reference SAgs. However, little is known about the precise amino acid position and/or composition responsible for TCR Vβ specificity in the C-terminal region. The MMTV SAgs, whose sequences are almost identical, often display slightly different TCR Vβ specificities due to differences in binding affinity and/or differences in expression levels [30]. The results from this study indicate that the majority of the SAg isoforms identified in this study display unique C-terminus region sequences and are different from the reference endogenous MMTV SAgs. This finding may suggest that the TCR Vβ specificity and binding affinity of some of these SAg isoforms may be altered, in part or full, due to changes in the interactions of the hypervariable C-terminus domain with the TCR Vβ region. On the other hand, changes in certain amino acids in the SAg C-terminus region may not affect TCR Vβ specificity at all [29]. It may be necessary to determine the TCR Vβ specificity of each MMTV SAg isoform by an *in vitro *T-cell activation study.

### *Mtv-8 *locus within the variable region of immunoglobulin κ chain on chromosome 6

**Figure 4:**
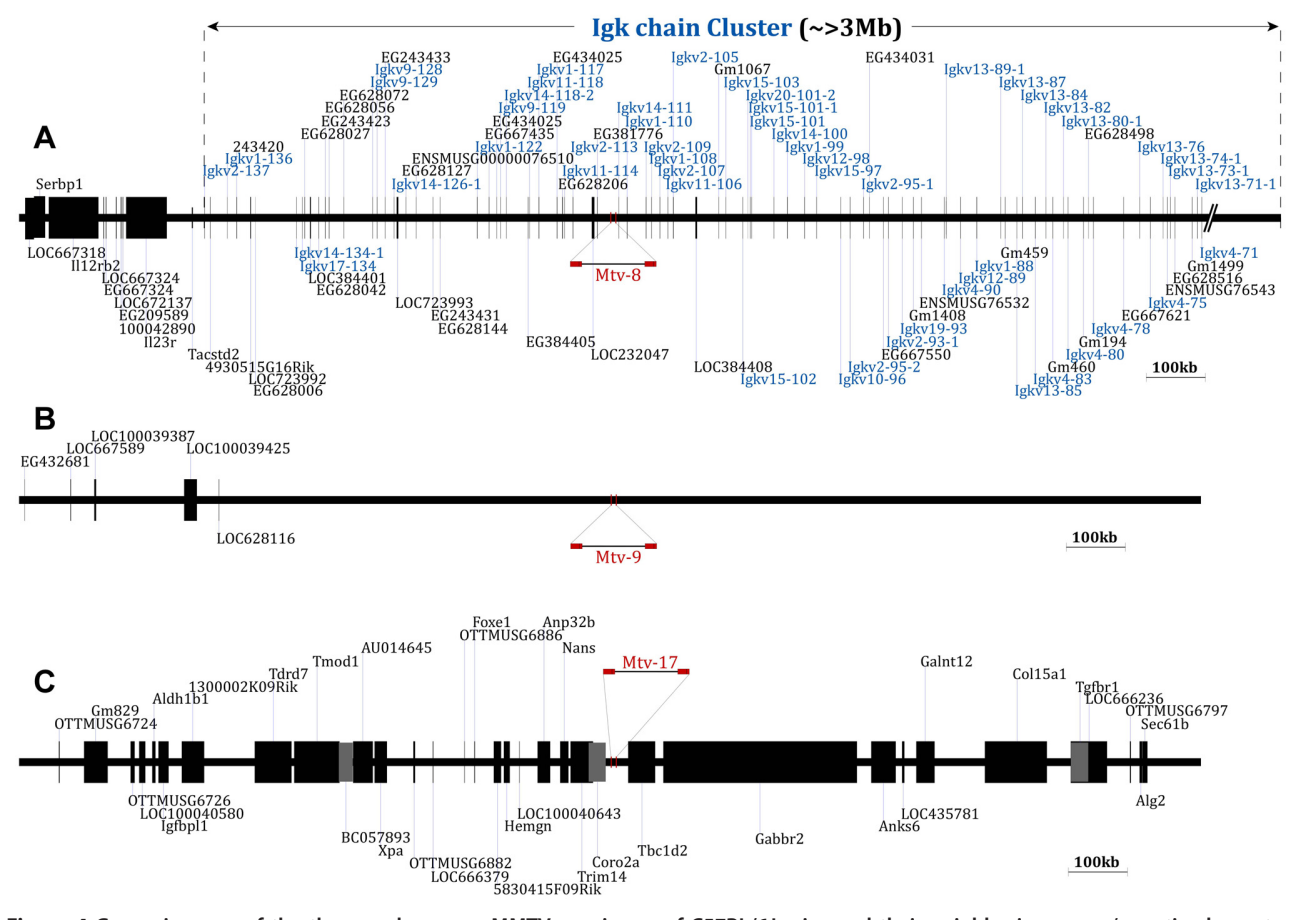
**Genomic maps of the three endogenous MMTV proviruses of C57BL/6J mice and their neighboring genes/genetic elements**. The loci of three endogenous MMTV proviruses (**A**: *Mtv-8*, **B**: *Mtv-9*, and **C**: *Mtv-17*) in the genome of C57BL/6J mice were mapped based on the NCBI mouse genome database and are schematically presented on a relative scale. The genes/genetic elements residing within regions upstream (~1 Mb) and downstream (1~2 Mb) of each *Mtv *locus were also mapped. The variable gene segments of the Igκ chain identified in this region are indicated in blue. Overlapping genes/genetic elements are differentiated with gray in the genomic map of *Mtv-17*.

In this study, three MMTV proviral loci (*Mtv*-8, *Mtv-9*, and *Mtv*-17) were mapped on the C57BL/6J genome based on the NCBI database. Detailed maps of the genes and other genetic elements surrounding the loci of *Mtv-8*, *Mtv-9*, and *Mtv-17*, were established by surveying sequences upstream (~1 Mb) and downstream (1~2 Mb) of each locus and are presented in Figure 4. As expected, there were only three endogenous MMTV loci (*Mtv-8 *[chromosome 6], *Mtv-9 *[chromosome 12], and *Mtv-17 *[chromosome 4]) in the genome of C57BL/6J mice and the *Mtv-8 *provirus was integrated into the variable region of immunoglobulin κ (Igκ) chain (Figure 4A). It needs to be noted that *Mtv-8 *has previously been mapped to this specific genomic region [34]. A very limited number of annotated genes/genetic elements were found in the region surrounding the *Mtv-9 *locus on chromosome 12 (Figure 4B). Annotated genes/genetic elements near the *Mtv-8 *locus on chromosome 6 of C57BL/6J genome are listed in Table 1.

**Table 1:** Annotated genes/genetic elements near the *Mtv-8 *locus on chromosome 6 of C57BL/6J genome

Gene ID	Location		Strand	Gene ID	Location		Strand
					
	Start - End				Start - End		
LOC667318	67202018 - 67217006		-	**Igκv2-109**	68252433 - 68253152		+
Serbp1	67216973 - 67235957		+	**Igκv1-108**	68262048 - 68262353		+
Il12rb2	67242012 - 67326131		-	**Igκv2-107**	68276092 - 68276746		+
LOC667324	67334405 - 67335335		-	**Igκv11-106**	68289521 - 68289989		+
EG667326	67339326 - 67339514		-	**Igκv2-105**	68298655 - 68299435		+
LOC672137	67356478 - 67356775		-	LOC384408	68339183 - 68336335		-
EG209589	67363870 - 67365006		-	Gm1067	68375599 - 68376066		+
100042890	67367652 - 67368047		-	**Igκv15-103**	68387462 - 68387919		+
Il23r	67372926 - 67441849		-	**Igκv15-102**	68416584 - 68416120		-
Tacstd2	67484053 - 67485816		-	**Igκv20-101-2**	68424882 - 68425092		+
**Igκ Cluster**	**67505630 - 70676748**			**Igκv15-101-1**	68429076 - 68429567		+
**Igκv2-137**	67505630 - 67506210		+	**Igκv15-101**	68431415 - 68431712		+
4930515G16Rik	67515693 - 67515228		-	**Igκv14-100**	68469006 - 68469471		+
**Igκv1-136**	67543870 - 67544302		+	**Igκv1-99**	68491652 - 68492416		+
243420	67559739 - 67560502		+	**Igκv12-98**	68520757 - 68521229		+
LOC723992	67584114 - 67582894		-	**Igκv15-97**	68541374 - 68541826		+
EG628006	67591690 - 67591491		-	**Igκv10-96**	68582424 - 68581959		-
**Igκv14-134-1**	67661622 - 67661168		-	**Igκv2-95-2**	68598525 - 68597993		-
**Igκv17-134**	67671219 - 67670723		-	**Igκv2-95-1**	68620746 - 68621329		+
EG628027	67674908 - 67675655		+	EG434031	68630373 - 68630842		+
LOC384401	67685575 - 67684065		-	EG667550	68654972 - 68654502		-
EG628042	67698717 - 67698518		-	**Igκv2-93-1**	68663502 - 68662919		-
EG243423	67709694 - 67710407		+	**Igκv19-93**	68686758 - 68686291		-
EG628056	67716030 - 67716766		+	Gm1408	68705587 - 68705032		-
EG628072	67741040 - 67741505		+	ENSMUSG76532	68719097 - 68718549		-
**Igκv9-129**	67789787 - 67790260		+	**Igκv4-90**	68757702 - 68757174		-
**Igκv9-128**	67797403 - 67797868		+	**Igκv13-89-1**	68760489 - 68760537		+
EG243433	67811153 - 67811653		+	**Igκv12-89**	68785301 - 68784840		-
**Igκv14-126-1**	67826005 - 67826471		+	**Igκv1-88**	68813025 - 68812259		-
LOC723993	67833604 - 67830690		-	**Igκv13-87**	68852795 - 68853260		+
EG628127	67846171 - 67846636		+	Gm459	68860932 - 68860405		-
ENSMUSG76510	67863567 - 67864046		+	**Igκv13-85**	68880728 - 68880263		-
EG243431	67892534 - 67892070		-	**Igκv13-84**	68889596 - 68890061		+
EG628144	67904684 - 67904224		-	**Igκv4-83**	68912013 - 68911372		-
**Igκv1-122**	67966736 - 67967482		+	**Igκv13-82**	68929192 - 68929657		+
EG667435	67986812 - 67987312		+	Gm460	68941288 - 68940752		-
EG434025	67999977 - 68000448		+	**Igκv13-80-1**	68959137 - 68959507		+
**Igκv9-119**	68006339 - 68006804		+	**Igκv4-80**	68967074 - 68966552		-
**Igκv14-118-2**	68016363 - 68016822		+	Gm194	68993499 - 68992966		-
EG384405	68053427 - 68053083		-	EG628498	69001439 - 69001788		+
**Igκv11-118**	68055408 - 68055874		+	**Igκv4-78**	69010218 - 69009684		-
**Igκv1-117**	68071085 - 68071819		+	EG667621	69061426 - 69060898		-
EG434026	68101879 - 68102611		+	**Igκv13-76**	69087860 - 69088326		+
EG628206	68110791 - 68111710		+	**Igκv4-75**	69106653 - 69106112		-
**Igκv11-114**	68114453 - 68114917		+	**Igκv13-74-1**	69127410 - 69127781		+
**Igκv2-113**	68128795 - 68129558		+	ENSMUSG76543	69135355 - 69134820		-
LOC232047	68165365 - 68160919		-	**Igκv13-73-1**	69140067 - 69140276		+
EG381776	68169975 - 68170699		+	EG628516	69148110 - 69147577		-
*Mtv-8*	*68192379 - 68193706*		+	Gm1499	69177377 - 69176848		-
**Igκv14-111**	68206398 - 68206863		+	**Igκv13-71-1**	69185921 - 69186152		+
**Igκv1-110**	68220521 - 68221259		+	**Igκv4-71**	69193682 - 69193154		-

The Igκ variable gene segments located within 1 Mb upstream and 1 Mb downstream of *Mtv-8 *locus are indicated in bold. The coordinates of the entire Igκ chain cluster are indicated (underlined).

It is documented that DNA sequences under the control of immunoglobulin gene promoters/enhancers are subjected to somatic hypermutation, which is frequently observed in the immunoglobulin variable gene segments after antigenic stimulation for a positive and/or a negative selection of developed B cells [35,36]. Based on the *Mtv-8*'s location in the variable region of Igκ gene cluster, we can speculate that the *Mtv-8 *proviral sequence is subjected to somatic hypermutations following antigenic stimulation of B cells, which is reflected in certain *Mtv-8*-derived SAg isoforms identified in this study. It will be interesting to investigate whether the *Mtv-8 *loci in other mouse strains, such as BALB/c and C3H, undergo similar somatic hypermutation events in comparison to the other *Mtv *loci, which are not embedded near the immunoglobulin clusters. In addition, the finding that the *Mtv-8 *provirus is integrated into the Igκ variable region suggests that it may be deleted from the genome depending on the structure of the variable regions of the individual chains during Igκ gene rearrangement in developing B cells. Thus, certain mature B cells may not be able to express the *Mtv*-8 SAg and/or its isoforms.

## Conclusions

It has been reported that a peripheral selection event involving dynamic and persistent induction of T cell tolerance configures the peripheral T cell repertoire [37]. A range of factors, including an individual's genetic profile and pathophysiologic status, may contribute to the process of shaping the peripheral T cell repertoire. The existence of a substantially diverse population of MMTV SAg coding sequences (both genomic and expressed isoforms) may be directly linked to the differential as well as dynamic shaping of the peripheral T cell repertoire in mice. On the other hand, incomplete or failed induction of tolerance against the freshly formed endogenous SAg isoforms may lead to local as well as systemic accumulation of autoreactive T cell populations.

## Methods

### Animal experiments

Female C57BL/6J mice from the Jackson Laboratory (Bar Harbor, ME) were housed according to the guidelines of the National Institutes of Health. The Animal Use and Care Administrative Advisory Committee of the University of California, Davis, approved the experimental protocol. Mice were sacrificed by cervical dislocation followed by tissue collection.

### Cloning of SAg coding sequences from genomic DNAs and cDNAs

Total RNA isolation and cDNA synthesis were performed based on the protocols described previously [38]. Briefly, total RNA was isolated from the tissues using the RNeasy kit (Qiagen, Valencia, CA). Total RNA (100 ng) samples were subjected to reverse transcription using Sensiscript reverse transcriptase (Qiagen). The sequence of the oligo-dT primer was as follows: 5'-GGC CAC GCG TCG ACT AGT ACT TTT TTT TTT TTT TTT T-3'. The genomic DNAs from bone marrow, liver, and lung tissues of normal mice were prepared using a DNeasy Tissue kit (Qiagen). A set of primers, MTV-1B (forward: 5'-TGC CGC GCC TGC AGC AGA AAT G-3') and MTV-2A (reverse: 5'-TGT TAG GAC TGT TGC AAG TTT ACT C-3'), was used to amplify the MMTV SAg region from the cDNA of normal tissues [39]. PCR using these primers was performed with the following conditions: hot start of 3 minutes at 94°C and 33 cycles of 94°C for 30 seconds, 55°C for 1 minute, and 72°C for 1 minute. Another set of primers, 5'-SAg (forward: 5'-CGG AAT TCC GAA AGG GGA AAT GCC GCG CCT-3') and 3'-SAg (reverse: 5-GAC GGC GGC CGC CCG CAA GGT TGG GCT CAT AA-3'), was used to amplify the SAg region from the genomic DNA of normal tissues. The following PCR condition was applied with these primers: hot start of 3 minutes at 94°C and 30 cycles of 94°C for 30 seconds, 50°C for 1 minute, and 72°C for 1 minute.

PCR products of the MMTV SAg regions were cloned into a pGEM-T Easy vector (Promega, Madison, WI). Sequencing was performed at the Molecular Cloning Laboratory (South San Francisco, CA). Sequences were trimmed for the SAg coding sequence before alignment and open reading frame (ORF) analyses using the Lasergene program (DNASTAR, Madison, WI). The following SAg clones were isolated from individual experimental groups and subjected to downstream analyses: 45 clones from genomic DNA-normal bone marrow, 30 clones from genomic DNA-normal lung, 23 clones from genomic DNA-normal liver, 23 clones from cDNA-normal bone marrow, and 4~6 clones from cDNA-all normal tissues except bone marrow.

### Phylogenetic analysis of SAg sequences

Phylogenetic analyses of the SAg coding sequences and translated sequences (both full-length and 74 amino acids of C-terminus hypervariable region) were performed using the neighbor-joining method within the MEGA4 program [40,41]. Bootstrapping was performed with 100 replications to evaluate the statistical confidence of branching patterns.

### Evaluation of putative TCR Vβ specificity of SAg isoforms

The C-terminus hypervariable regions (~74 amino acids) of the individual SAg isoforms were compared with the same regions of the reference SAg sequences of which TCR Vβ specificities were previously defined using the Lasergene program (DNASTAR) [5,11,20,42,43]. The percentage similarity of the C-terminus regions of the SAg isoforms to the references was calculated.

### Mapping of MMTV proviruses and their neighboring genes/genetic elements

Endogenous MMTV proviruses residing on the genome of C57BL/6J mice were mapped by a BLAST search of the National Center for Biotechnology Information (NCBI) database using the SAg sequences of *Mtv-8*, *Mtv-9*, and *Mtv-17 *as probes [31,44]. In addition, the genes/genetic elements neighboring the individual proviral loci were mapped by surveying the genomic region within 1 Mb upstream and downstream regions using both the Ensembl and NCBI genome databases.

## Authors' contributions

This study was conceived, designed, and managed by KC. DGG participated in active scientific discussion. YKL, SC, and AC participated in the collection and analysis of the results, and YKL and SC finalized the figures. YKL also contributed to the drafting of the manuscript. All authors read and approved the final manuscript.
